# The Relationship between Disturbed Sleep, OSAS, and Metabolic Diseases

**DOI:** 10.1155/2019/1463045

**Published:** 2019-08-04

**Authors:** Patrizio Tatti, Abd Tahrani, Desiderio Passali, Sirimon Reutrakul, Thirumagal Kanagasabai

**Affiliations:** ^1^Department of Endocrinology and Diabetes, INI Institute, Rome, Italy; ^2^Institute of Metabolism and Systems Research, University of Birmingham, Birmingham, UK; ^3^ENT Department, University of Siena, Italy; ^4^Division of Endocrinology, Diabetes and Metabolism, Department of Medicine, University of Illinois at Chicago, Chicago, Illinois, USA; ^5^Department of Epidemiology, Biostatistics and Occupational Health, McGill University, Montreal, Quebec, Canada

The relationship among sleep quality, duration, regularity and metabolic disorders is an unknown territory. We know that sleep has its own architecture, made of progressive stages, recurring throughout the night, but we largely ignore the meaning of this, and which consequences the disruption of this architecture may cause. We do not even know what the optimal duration of sleep is, a topic that has been debated since the Epic of Gilgamesh. It is only in the final years of the last century that we could gain some insight into the medical meaning of sleep and the consequences of its disruption. Being the most obvious marker and cause of disturbed sleep, OSAS has come in the crosshair of most researchers.

We know that OSAS/disturbed sleep has a causative role in most metabolic and cardiovascular disorders and possibly even in cancer.

This issue of the journal has the merit of bringing together the best answers available to, at least, some of the mysteries surrounding sleep.

The paper from W. Martorina and A. Tavares, “Real-World Data in Support of Short Sleep Duration with Poor Glycemic Control, in People with Type 2 Diabetes Mellitus,” on 140 50- to 61-year-old patients examines the effect of the “sum up sleep,” thus encompassing both night- and daytime sleep segments. This aspect is of special interest because before the advent of artificial light people used to sleep in two segments, interrupted by two or three hours of wakefulness, during which they attended religious practices or social events. This paper supports the idea that the final outcome on the HbA1c level does not dramatically change if we add this “catch up sleep.” In most countries, people used to nap in the afternoon, and we had previously no insight on the effect of this practice.

The paper from K. Neumann et al., “Sleep-Disordered Breathing Is Associated with Metabolic Syndrome in Outpatients with Diabetes Mellitus Type 2,” uses the severity of OSAS to assess the relationship with the metabolic syndrome in subjects with type 2 diabetes mellitus. One of the merits of this study is the demonstration of a “dose-dependent effect,” one of the most relevant of the Bradford-Hill criteria used to accept statistical results.

The study from Y. Zhang et al. adds an in-depth study of glucose metabolism. Using the HOMA Index and the overnight metabolic profile, the authors could demonstrate a change in the indexes of insulin resistance, a deep involvement of the hypothalamic-endocrine axis and an increase of the inflammatory cytokines in the most severe OSAS patients.

The paper by J. Zou et al. entitled “The Relationship between Simple Snoring and Metabolic Syndrome: A Cross-Sectional Study” assessed the cross-sectional relationship between simple snoring and metabolic syndrome in a sample of 866 adults from southeastern China without diagnosed sleep breathing disorders, such as apnea and hypoxia. This study is unique in several ways, including its study population, a thorough analysis of the multiple metabolic outcomes, and its evaluation of gender-specific effects. The overall findings not only suggest that simple snoring is independently linked to metabolic syndrome but also that snoring severity linearly relates to metabolic score and that females are more vulnerable to metabolic disorders. On gender-specific effects, snoring was linked with hypertension in males and abdominal obesity and dyslipidemia in females, highlighting the need for additional research to develop gender-specific prevention, management, and targeted intervention strategies [1]. However, this early paper on free-living adults without diagnosed sleep breathing disorders was slightly weakened by its use of bedpartner-reported snoring data whereas even the use of noninvasive objective measurement tools to measure snoring may yield somewhat imprecise results among participants with bedpartners [2].

The paper by Y. Liu et al. entitled “Effect of the Interaction between Obstructive Sleep Apnea and Lipoprotein(a) on Insulin Resistance: A Large-Scale Cross-Sectional Study” assessed the interaction effect of obstructive sleep apnea and serum lipoprotein(a) on insulin resistance (i.e., HOMA-IR) in 4,152 participants from the Shanghai Sleep Health Study cohort. The findings support an inverse relationship between serum lipoprotein(a) and the severity of insulin resistance, and in lower serum lipoprotein(a) levels, the relationship between obstructive sleep apnea severity and insulin resistance was even stronger. Given the mounting evidence suggesting a global rise in sleep disorders and cardiometabolic diseases, the findings from this study offer a unique perspective on the complicated relationship between serum lipoprotein(a) and insulin resistance in obstructive sleep apnea [3–5]. Clinically, this study, however, raises an important question: what is the optimal lipid-lowering medication level that is associated with the lowest insulin resistance risk in patients with obstructive sleep apnea? Further research is also needed to elucidate whether these effects are different in various groups (i.e., gender, age, ethnicity, and comorbidities).
